# Association of Age and Temperamental Traits with Children’s Behaviour during Dental Treatment

**DOI:** 10.3390/ijerph19031529

**Published:** 2022-01-29

**Authors:** Maria Lilia Adriana Juárez-López, Miriam Marin-Miranda, Jesús Lavalle-Carrasco, Alberto Pierdant, Leonor Sánchez-Pérez, Nelly Molina-Frechero

**Affiliations:** 1 Department of Paediatric Dentistry, FES Zaragoza, National Autonomous University of Mexico (UNAM), Mexico City 09230, Mexico; liadju@yahoo.com (M.L.A.J.-L.); miriam.marin@zaragoza.unam.mx (M.M.-M.); 2 Department of Health Care, Division of Biological and Health Sciences, Autonomous Metropolitan University (UAM), Mexico City 04960, Mexico; lavallec@outlook.com (J.L.-C.); aipierdant@gmail.com (A.P.); tlsperez@correo.xoc.uam.mx (L.S.-P.)

**Keywords:** temperament survey, children’s behaviour, dental treatment, cooperation

## Abstract

During paediatric dental treatment, cooperation by children is essential, and temperament can determine their behaviour style. This study aimed to associate temperamental traits and age with behaviour during paediatric dental treatment. This was an observational and cross-sectional study of patients aged 3–10 years. To determine the temperamental traits, an Emotionality Activity and Sociability Temperament Survey (EAS) was performed with the children’s parents. The type of behaviour was determined with Frankl’s criteria. The total scores for temperamental traits, means, and standard deviations were obtained. The chi-squared test, one-way ANOVA and Student’s t-test were applied. A total of 140 patients, who were 5 ± 2.1 years old, participated in the study. Age was determinant for negative behaviour, with a greater frequency in children ≤5 years old (*p* < 0.05), and a significant negative correlation between age and behaviour (rho = −0.245, *p* = 0.001). Patients with higher emotionality and activity presented negative and definitely negative behaviours more frequently (*p* < 0.01). The values for sociability and shyness were similar for positive or negative behaviours. In conclusion, the temperamental traits of emotionality and activity were more frequent in children with disruptive behaviours; therefore, conducting a survey on temperamental traits can be useful for planning behavioural approaches in paediatric dentistry.

## 1. Introduction

The cooperation of children during treatment is one of the main challenges of paediatric dental care; hence, analysing the factors that influence the behavioural responses of children is important. The behaviour of children is determined by innate and learned factors: cognitive development, personality, maturity, age, and experiences. It is also related to medical history, the attitude of the parents, and the office environment, among other factors [1,2].

The reaction of children to the same stimulus can be different for each child and is based on their personality, which includes the set of physical, mental, and emotional characteristics, habits, and traits that make each individual different. Personality is constituted by character and temperament. Character refers to the traits that are shaped by the processes of cognitive development and life experiences that influence feelings, behaviour, decisions, judgements, and orientations. Character has three dimensions: cooperation, self-transcendence, and self-direction. These dimensions are influenced by the sociocultural and family environment, and the relationship with the environment [3].

Temperament refers to the innate psychological dispositions that determine the behaviour of an individual in the face of external and internal stimuli, that is, their behaviour style [4,5]. The evolutionary temperamental theory by Buss and Plomin states that temperament traits are of genetic origin and appear in infancy [5]. However, temperament does not remain static for life, and can change during the development and maturation of children due to their interactions with other people as well as their environment [6,7].

Rothbart et al. noted that temperament is a consequence of reactivity and self-regulation [8]. Reactivity is the expression of emotions, which is usually different in each individual in relation to the following:

- The intensity of the stimulus, such as unpleasant or painful acts during a dental consultation that cause negative reactions in children;
- The novelty of the stimulus, such as first consultations and separation from the parents leading to distrust;
- The internal condition of the individual at the biological and/or psychological level, as well as elements of a calming nature that are present, for example, when children hear the voice of their mother or the use of music therapy for stress control.

Knowing temperamental traits can be useful as a predictor of behaviours and psychological problems in children; therefore, different instruments have been developed and used [9]. The Emotionality Activity and Sociability Temperament Survey (EAS) is based on an analysis of inherited traits of early appearance and has been applied from early infancy to adulthood [10]. There are reports of the application of the EAS in paediatrics, anaesthesiology, and neurology, but its application in the field of dentistry is scarce.

In children, the EAS is considered to have satisfactory internal consistency and has been used to analyse their temperamental traits [10].

Activity refers to motor function, sleep cycles, wakefulness, and periods of inactivity, stillness, or hyperactivity. Emotionality is understood as the energy and intensity of a reaction to a stimulus and the ability to adapt to certain situations through acceptance (positive, e.g., smiling) or rejection (negative, e.g., isolation, crying, or tantrums). Sociability is manifested by the interaction, communication, empathy, or shyness capacity of a child [10].

For providing paediatric dental care, the behavioural approach of the dentist is important; therefore, having an instrument that investigates the temperamental traits of children can be useful to predict their behavioural reactions during treatment. Hence, the aim of this study was to determine whether temperamental traits and age are associated with behaviour and the usefulness of the EAS survey to recognize traits that cause dysfunctional behaviours during paediatric dental treatment.

## 2. Materials and Methods

### 2.1. Study Population

This is a cross-sectional study conducted in the clinic “Reforma” of the FESZ, National Autonomous University of Mexico (UNAM). All the paediatric patients aged 3 to 10 years who attended for dental treatment from August 2019 to January 2020 were invited to participate. Patients with asymptomatic dental caries and without previous dental treatment experience were included in this study. Those that required emergency treatment due to episodes of acute pain or active infection, and with an indication of dental extraction due to severe dental caries, were excluded. Another exclusion criterion was children whose mothers or guardians were not able to attend the interview. With a descriptive purpose, the age and gender of the participants were recorded.

### 2.2. Ethics

The protocol complied with the Declaration of Helsinki guidelines and the guidelines of the faculty research committee, and was approved by the Ethics Committee of the university (IRB: 20/13, 10/18, CE.012.18). The parents or guardians of the children were informed about the objectives of the study, and only those children whose parents provided their consent by signing an informed consent form and responding to the survey were included. To avoid bias and to maintain consistency regarding the use of observation only during treatment, no questions were asked to the children; they were only observed.

### 2.3. Interview

A questionnaire written in Spanish was used to determine the temperamental traits. The patients’ age and gender were recorded. The temperament survey was answered by the parents or guardians of the child and was structured based on the EAS [10], with 4 domains for temperament: emotionality, activity, sociability, and shyness. Before performing the study, to evaluate the clarity of the questions, a pilot study was previously conducted with 10 parents that were not included in this research. Interviews were performed face-to-face by one trained examiner before the appointment of the treatment, with a duration of 15–20 min.

### 2.4. Dental Treatment

Treatments were provided by postgraduate residents who applied behavioural guidance techniques, such as tell–show–do, modelling, and positive reinforcement. These techniques were routinely used in paediatric patients to encourage them to cooperate. The behaviour was assessed in the second appointment of the treatment to remove dental caries and for restorative procedures of one or two molars affected by dental caries under infiltrative anaesthesia and by using a rubber dam for dental isolation. The restorations performed were composites and stainless-steel crowns without pulpal therapy. The treatments lasted from 15 to 40 min. The parents were always present at the procedure, but did not participate in the management of the child.

To determine behaviour type, a single clinician who specialized in paediatric dentistry applied the Modified Frankl scale. The intra-examiner agreement (Kappa) for this clinician was 0.79. Additionally, this clinician did not have access to the results of the temperament questionnaire, nor did he participate in the treatments. The assessment of behaviour type was always performed at the second appointment.

Based on the behaviour presented, the children were classified as definitely negative, negative, positive, and definitely positive, for which the presence of crying, disruptive movements, rejection, and cooperation was used as a criterion (Table 1) [11,12].

### 2.5. Data Analysis

The responses were scored using a Likert scale, with a numerical value assigned to each response option (never, 0; rarely, 1; sometimes, 2; frequently, 3; and always, 4) [13].

Each domain was evaluated through 5 questions or items, and there was a total of 6 inverted items (Table 2).

The inverted questions were subtracted from the sum of the domain in which they were included, considering the following formula proposed by Likert:

Pi = (Pm + 1) − Po(1)

where:

- Pi: transformed score for the inverted item;
- Pm: maximum score for the item;
- Po: original score for the inverted item.

For the data analysis, the frequencies, percentages of the behaviours presented based on sex and age group, and measures of central tendency, such as the means and standard deviations, were calculated, as were the means for each temperament domain. The chi-squared test was applied to compare groups with qualitative values, ANOVA and the Student’s t-test were used to compare groups with quantitative variables, and the Spearman’s rank correlation coefficient test was applied. Data were coded and analysed using SPSS statistics software 27.0.0 (IBM, Armonk, NY, USA), and an alpha level of <0.05 was considered statistically significant.

## 3. Results

A total of 140 patients (5 ± 2.4 years of age) participated in this study; 42% were male, and 58% were female. When analysing the behaviour presented during dental treatment, no difference was observed with respect to sex. The distribution of the behaviour by age is presented in Table 3, showing a significant negative correlation between age and behaviour (rho = −0.245, *p* = 0.001).

When the results were grouped into preschoolers and schoolchildren, it was found that children ≤ 5 years of age presented negative behaviours more frequently (*p* = 0.026).

When analysing temperamental traits by type of behaviour, children who presented negative behaviours had higher emotionality and activity values than children who exhibited positive behaviours (*p* < 0.05) (Table 4).

When the results were grouped into positive behaviour (positive and definitely positive) and negative behaviour (negative and definitely negative), a statistically significant difference in the emotionality (*p* = 0.01) and activity (*p* = 0.001) dimensions was found. The values for sociability and shyness were similar for children who exhibited positive or negative behaviours (2.2 and 2.7, respectively). By comparing the scores of the temperamental traits between the groups of preschoolers (3–5 years old) and schoolchildren (6–10 years old), a difference within the activity and sociability domains was observed (Table 5).

## 4. Discussion

To achieve optimal and quality dental treatment in paediatric patients, acceptance and cooperation by the patient is necessary; therefore, when planning a treatment approach for paediatric patients, having diagnostic elements allows clinicians to predict the behaviour of a child and select an effective communication strategy, behavioural guidance technique, or pharmacological technique, such as premedication or general anaesthesia. In this study, when applying the EAS, the values for emotionality and activity were higher for children who presented negative and definitely negative behaviours, a finding similar to that reported by Aminabadi et al., who indicated that temperament is a predictor of child behaviour in a dental office [14]. On the other hand, in the study by Jain et al., a low association between temperament and behaviour was found, reporting that emotionality and shyness may have additive effects over dental anxiety, and considering age as a moderator of temperament, since children learn to regulate their emotions as a result of the development of cognitive abilities [15].

In this study, activity was the temperamental trait that most frequently characterized patients with negative behaviours. An association has been noted between high activity domain scores and a high level of anxiety during the preoperative period, a finding that can be linked to problems regarding adapting to new environments, hyperactivity, and, in some cases, aggressiveness [16].

Children with emotionality traits exhibit greater hypersensitivity to stimuli, lower tolerance to noise, and intense reactions, which can be explosive [7]. The above explains why children with higher emotionality trait values presented negative or definitely negative behaviours, a finding similar to that reported by Arnrup et al., who related negative emotionality with disruptive behaviours in dentistry [17]. Another study reported that emotionality was a determinant for the presentation of fear and anxiety during the induction of general anaesthesia [18]. A study on preschoolers associated negative emotionality with aggressive behaviour problems, and the sociability trait with adaptation to new experiences [19].

Notably, emotional responses can be modified by other factors, such as the type of parenting and the development of capacities to modify emotions. Children with negative temperaments can learn to modify their reactions through support, attention, and boundaries; therefore, to reduce negative behaviours during dental treatment, it is advisable to establish communication with paediatric patients and clearly describe what is expected of them, thus avoiding confrontations. Additionally, strategies such as progressive desensitization, so as to gradually introduce children to the dental field and favour their adaptation, should be adopted [2].

Parents transmit to their children the fear of pain, leading to disruptive behaviours. Parents’ fear of injections has been related to the anxiety that their children present in the dental office [20]. Another study found that overindulgent parents induce capricious and apprehensive behaviours in their children. Authoritarian parents cause states of high anxiety and behavioural problems in their children [21]. Hence, it is important to recognize at the first appointment the temperamental traits of paediatric patients, as well as the family contexts of the children, that influence behavioural reactions during paediatric dentistry consultations.

As expected in this study, behaviour was correlated with age, where negative behaviours were more frequent in younger patients. During early infancy, there are fears specific to this age towards unknown situations; cognitive capacity in regard to establishing interpersonal relationships and self-regulation is limited. Young children are more vulnerable and restless due to a greater attachment to their mother, which partially explains their negative responses to dental treatment [18]. In this study, a higher activity trait was observed in younger children, while sociability was lower; both characteristics had an influence on negative behaviour and explain their lower adaptation towards dental treatment. Another study reported that 8–12-year-old children with difficult temperaments presented greater anxiety at the dental office, along with the presence of negative behaviour [22].

Importantly, fear during dental consultations also depends on the combination of other variables, such as the type of treatment and equipment used. The noise of a hand tool, anaesthetic puncture, and isolation with a rubber dam are known as triggers of fear [23]. Another factor is caries severity. Other factors associated with negative behaviour are sociocultural background and negative experiences regarding any type of health care [24]. Children with negative behaviours unfortunately have higher prevalence and severity of caries decay, further complicating the approach used by clinicians due to the more complex treatment requirements [25]. A higher risk of dental caries in early childhood was reported in children with negative temperamental traits [26]. Fear is one of the main reasons to avoid dental treatment and to postpone dental care. In this context, children with elevated emotive reactions present a lack of adaptation to situations of anxiety, such as dental attention [27].

The behavioural responses of children are more positive when minimally invasive treatments are applied than when dental treatments are prolonged and unpleasant [28]. To provide dental care to patients with negative temperaments, the approach to the dental environment must begin at an early age using techniques such as tell–show–do, positive reinforcement, modelling, and audio-visual distraction, which promote favourable behavioural changes and the cooperation of paediatric patients [29,30,31].

Finally, in this study, the application of a survey was useful as a predictor of paediatric dentistry patient behaviour; through such surveys, paediatric dentists can predict possible behavioural responses and thus improve the quality and warmth of clinical care.

The factors that have an influence on disruptive behaviours at the dental office are diverse; thus, it would be interesting to determine the relationship between the oral health status of the child and other aspects of the personality, such as temperamental traits. In this research, the type of performed dental treatment was unified; however, one of the limitations of this study was not having epidemiological data regarding the number of dental caries by child or information about their quality of life, aspects that should be considered in further research. Other limitations of this study that may influence the results obtained are the sincerity of the information provided by the mothers, as well as the differences in the time of dental treatments applied. The present results should be considered only in the context of the conditions that were evaluated, since this study was conducted in a community with several oral health issues and with restricted accessibility to dental care. Thus, the findings cannot be generalised to children that periodically receive dental care or have different treatment needs.

To analyse with greater precision the personality factors involved in the behaviour of children during dental treatments, we recommend considering, in addition to the knowledge of temperamental traits addressed in this study, aspects such as parental anxiety and the self-control of the paediatric patient.

## 5. Conclusions

In this study, through the EAS, it was found that a sum greater than 13 points for sociability and emotionality was a determinant of the presentation of negative behaviours in paediatric patients during dental treatment, and that age equal to or less than 5 years was associated with the presentation of negative behaviours.

In addition to personality traits, the behaviour of children during dental treatment is related to various factors, such as the influence of parental personality and the type of dental treatment. It is also related to the assertive communication skills, behavioural guidance, and dentist skills.

## Figures and Tables

**Table 1:** Modified Frankl scale.

I. Definitely negative	Refusing to play a game, crying forcefully or fearfully, or any other overt evidence of extreme negativism.
II. Negative	Reluctance to play, uncooperative behaviour, and some evidence of negative attitude that is not pronounced.
III. Positive	Acceptance of playing, willingness to comply with the dentist, cooperative behaviour.
IV. Definitely positive	Good rapport with the dentist, interested in the environment, laughing and enjoying the situation.

**Table 2:** Items per domain of the temperamental traits survey.

Activity	Emotionality	Sociability	Shyness
Always moving	Cries easily	Like to be with people	Has a tendency to be shy
When goings from one place to another, does it slowly *	Has a tendency to be emotional	Finds people more stimulating than anything else	Makes friends easily *
Since waking up in the morning, does not stop running	Protests and cries often	Prefers to play with other children than to play alone	Finds it difficult to trust strange people
Is very energetic	Easily upset	Is lonely *	Is very friendly with strangers *
Prefers games that are quiet and not active *	Reacts very strongly when upset	When alone, feels isolated	Is very sociable *

* Inverted items.

**Table 3:** Relationship of the patients’ age with the behaviour presented during dental treatment.

Years Old	Definitely Negative		Negative		Positive		Definitely Positive		Total	
	X	%	X	%	X	%	X	%	X	%
3	14	10	13	9.3	7	5	0	0	34	24.3
4	5	3.6	6	4.3	13	9.3	5	3.6	29	20.7
5	3	2.1	5	3.6	8	5.7	4	2.9	20	14.3
6	0	0	8	5.7	7	5	0	0	15	14.3
7	3	2.1	5	3.6	3	2.1	1	0.7	12	8.6
8	2	1.4	1	0.7	5	3.6	3	2.1	11	7.9
9	0	0	0	0	3	2.1	4	2	7	5
10	1	0.7	1	0.7	7	5	3	2.1	12	8.6
Total	28	20	39	27.9	53	37.9	20	14.3	140	100

Spearman’s rank correlation coefficient: rho = −0.245, *p* = 0.001.

**Table 4:** Temperamental traits with the children’s behaviour during dental treatment.

	Definitely Negative		Negative		Positive		Definitely Positive		*p* ^†^
	N 33		N 34		N 41		N 32		
	X	SD	X	SD	X	SD	X	SD	
Activity	15.9	±2.1 ^a,b^	15.5	±2.6	14.5	±2.9	14.2	±2.9	0.018
Emotionality	13.2	±2.9 ^c,d^	12.3	±2.9	11.1	±2.2	11.2	±2.9	0.006
Sociability	14.5	± 2.5	14.4	±2.5	13.8	±2.5	13.8	±3.2	0.672
Shyness	11.2	± 2.3	11	±1.4	11.4	±1.6	11.1	±1.5	0.895

Averages of the survey values of temperamental traits. † Variance test of an intergroup factor. Dunnett’s test. ^a^ Definitely negative versus definitely positive: *p* = 0.015; ^b^ definitely negative versus positive: *p* = 0.004; ^c^ definitely negative versus definitely positive: *p* = 0.022; ^d^ definitely negative versus positive: *p* = 0.051.

**Table 5:** Temperamental traits by age group.

	3–5 Years Old		6–10 Years Old		*p*-Value
	N = 91		N = 49		
	X	SD	X	SD	
Activity	15.3	2.4	14.3	2.7	0.029 *
Emotivity	11.9	2.7	11.8	3.1	0.81
Sociability	13.7	2.7	14.7	2.6	0.045 *
Shyness	11.1	2.0	11.4	1.9	0.30

* Student’s *t*-test.

## Data Availability

The datasets generated and/or analysed during the current study are available from the corresponding author upon reasonable request.
